# Supplementary material for: Westdrive X LoopAR: An Open-Access Virtual Reality Project in Unity for Evaluating User Interaction Methods during Takeover Requests
Source: Sensors (Basel). 2021 Mar 8;21(5):1879. doi: 10.3390/s21051879 (PMC7962530; doi:10.3390/s21051879)
Supplement: Supplementary file 1 [file sensors-21-01879-s001.pdf]

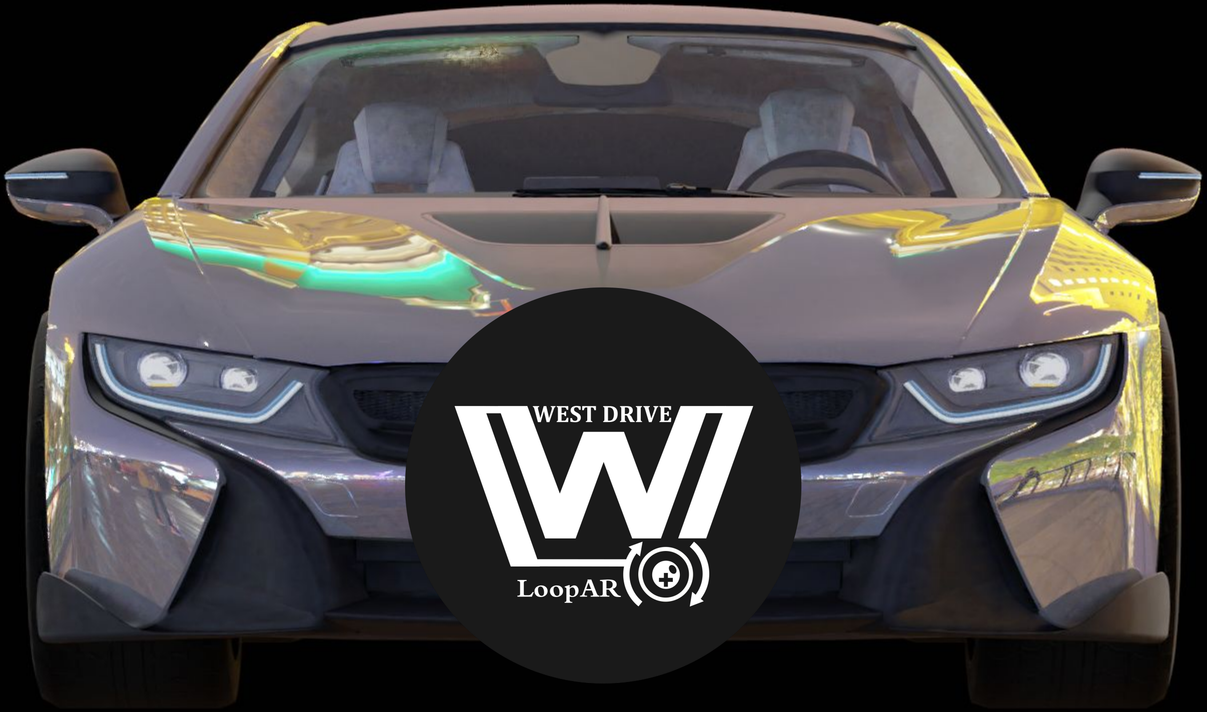

unity

a new approach for human-machine
interaction research for closing the out-of-the-loop unfamiliarity

blender

Virtual Reality

We are using virtual reality headsets for a high presence perception and the psychological sensation of being in an alternate space.

The HTC Vive Pro comes with the Tobii Eye Tracking system and allows monitoring the eye movement of the participant.

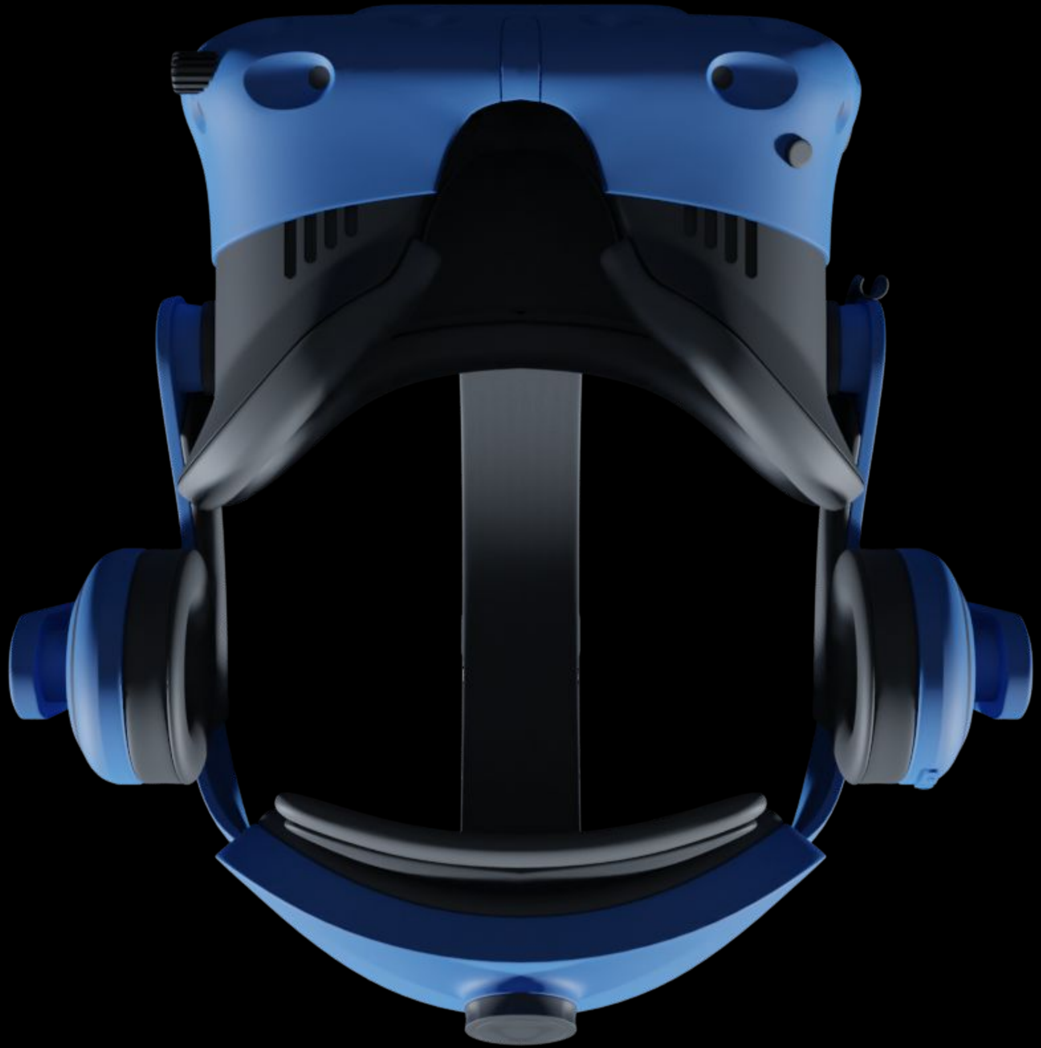

All components and functionalities working together in the Unity3D game engine with different modules for traffic behavior.

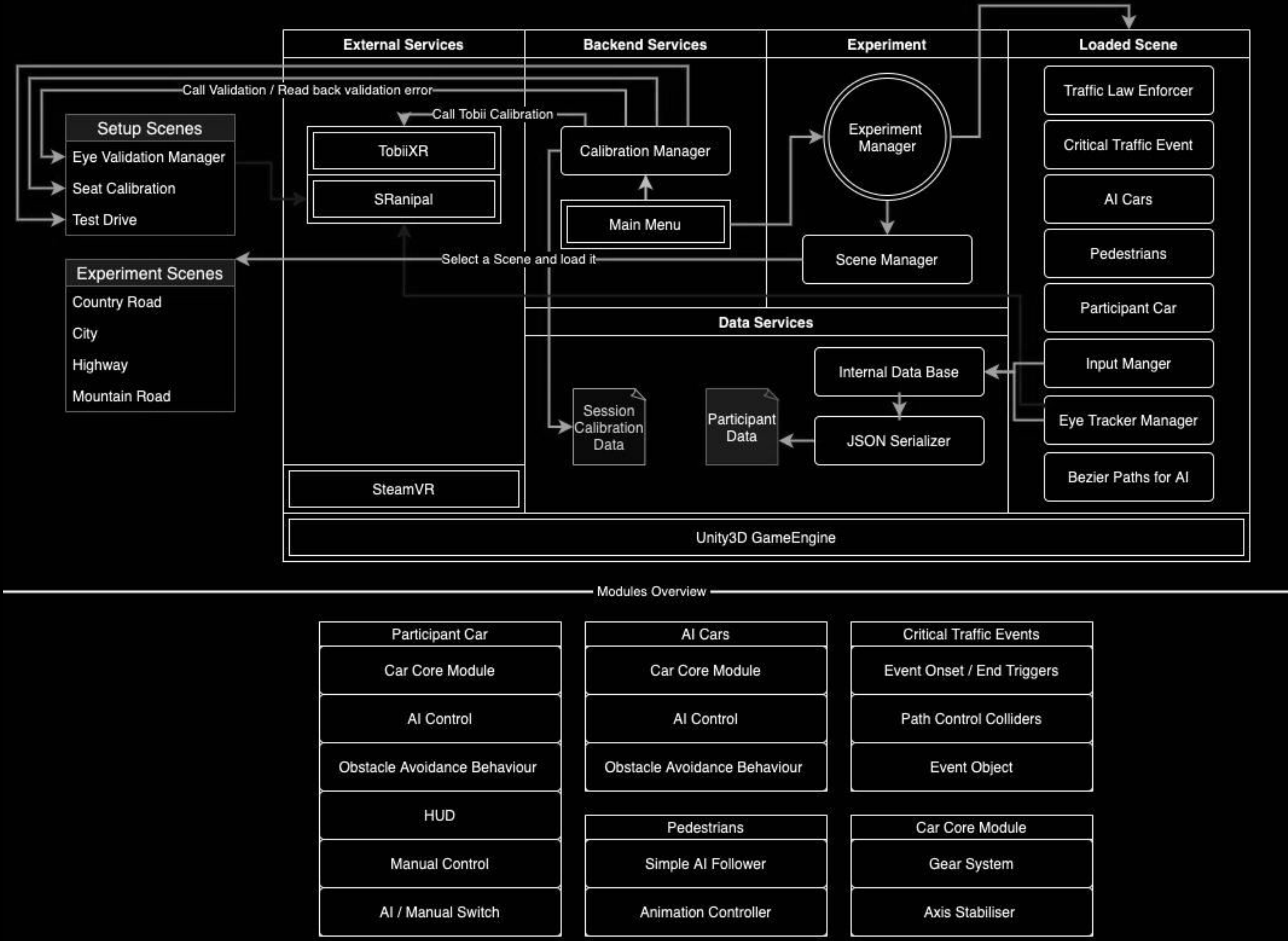

Level of detail (LOD)

One of the most common techniques for increasing hardware performance. Based on the ratio of the objects screen space height to the total screen height, different LODs will be activated. The objects geometry will be reduced in relation to the participant distance.

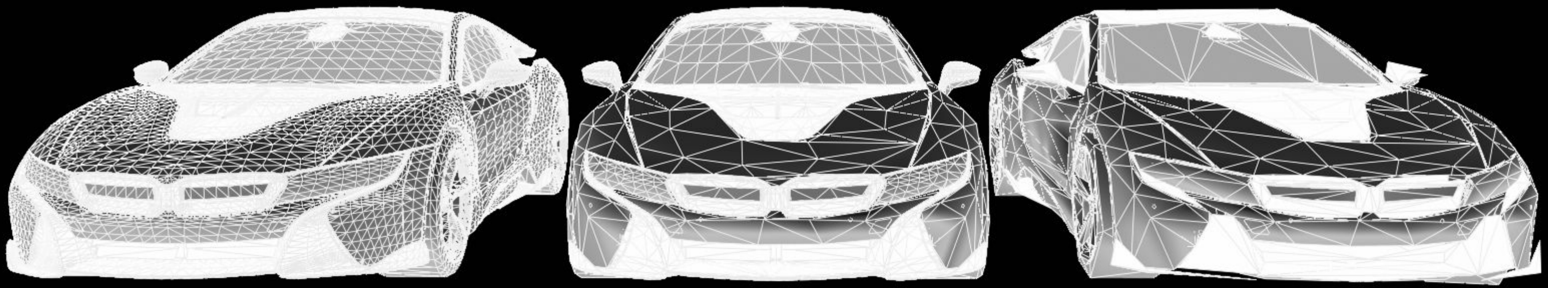

LOD_0
80k vertices

LOD_1
40k vertices

LOD_2
10k vertices

All pedestrians were created with Adobe Fuse Character Creator and animated with the Mixamo motion capture library.

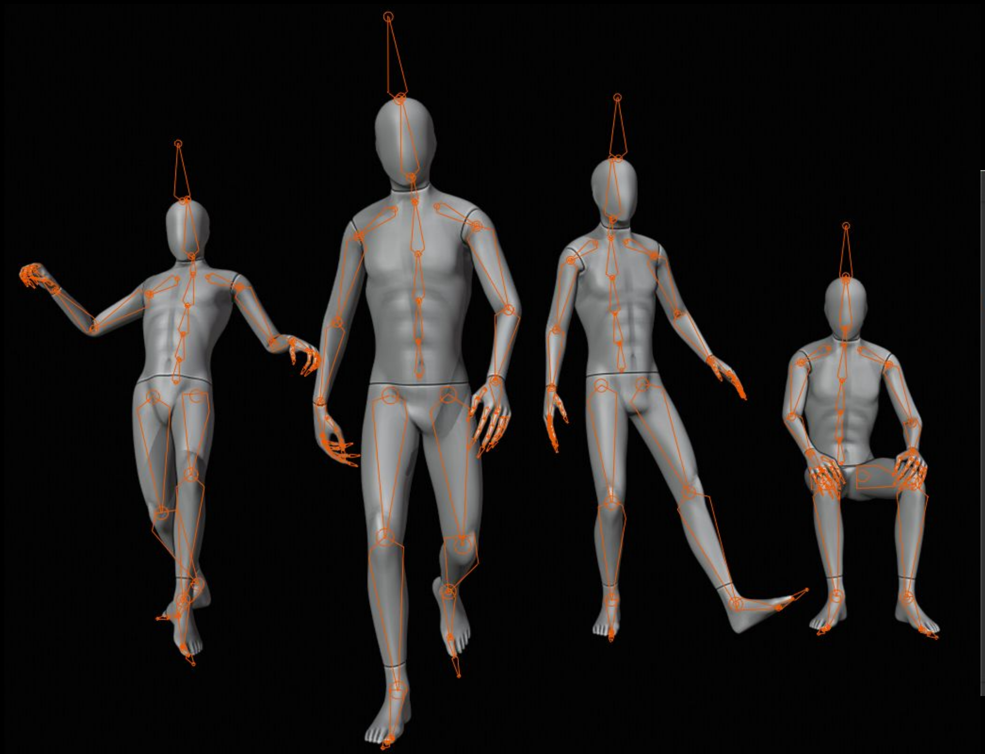

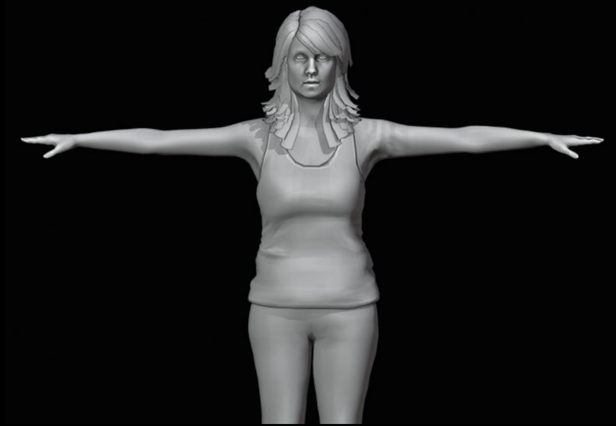

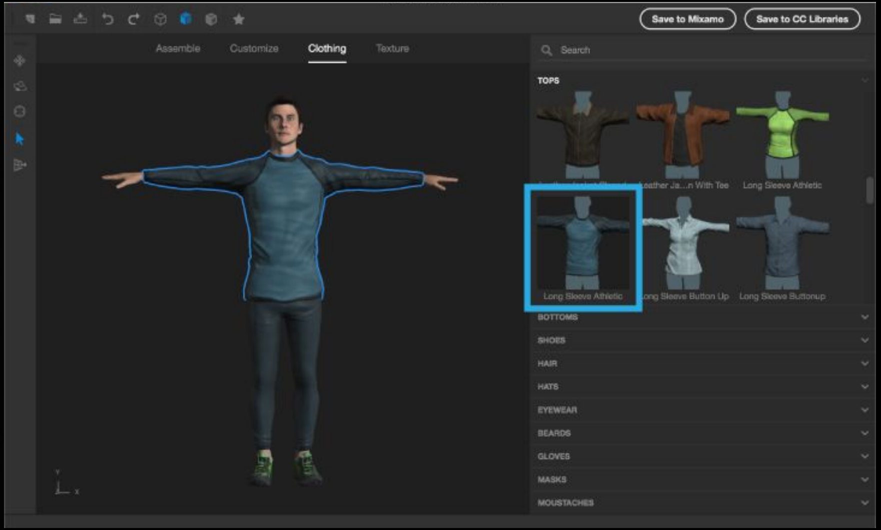

Adobe Fuse CC editor

List of assets

Asset Name	Link on Asset Store	Description	Paid / Free	Essentail / Optional
+ SteamVR	SteamVR Plugin	main api to use HTV Vive/ Vive Pro HMDs in Unity3d	free	note Essential
* SS07	Unlock super sport car #07	one of the car assets used in the project	paid	note Optional
* EasyRoads3D v3 pro	EasyRoads3D v3 pro	Create unique road networks directly in Unity with both built-in customizable dynamic crossing prefabs and custom crossing prefabs based on your own imported models	paid	note Essential
Conifers [BOTD]	Conifers	his package contains 4 conifers derived from Unity's Book of the Dead Demo – reworked, optimized and imported using the Custom Tree Importer to make them compatible with the "legacy" rendering pipeline.	free	note Optional
Standard Assets	Standard Assets	This collection of assets, scripts, and example scenes can be used to kickstart your Unity learning or be used as the basis for your own projects.	free	note Optional
Grass Flowers Pack Free	Grass Flowers Pack Free	This pack contains 12 grass and flower textures(Resolution 1024x1024,Alpha Channel).	free	note Optional
Rocky Hills Environment - light pack	Rocky Hills Environment - light pack	High quality, low poly model pack, good for any kind of platform, mobile friendly and very aesthetic for higher quality game development. Includes models from a early project. Each model from the Legacy project comes with 2 Lod levels.	free	note Optional
Terrain Tools Sample Asset Pack	Terrain Tools Sample Asset Pack	The Terrain Tools Sample Asset Pack contains a collection of Assets to jump-start development for users interested in utilizing Unity's growing Terrain system.	free	note Optional
Farm Machinery	Farm Machinery	This is a low poly style model pack of a farm tractor and a planter.	free	note Optional
Wooden Box	Wooden Box	This package contains a wooden box in four states: One intact and three destroyed versions.	free	note Optional

With a high variety of graphical assets we build an authentic environment for collecting scientific data.

The Unity Store and the platform SketchFab provide a high variety of free 3D models for all different kinds of virtual experimental setups.

Large terrain with continues road network of the length of roughly 11 kilometers

Main terrain includes a city, a mountain road, a country road and a highway

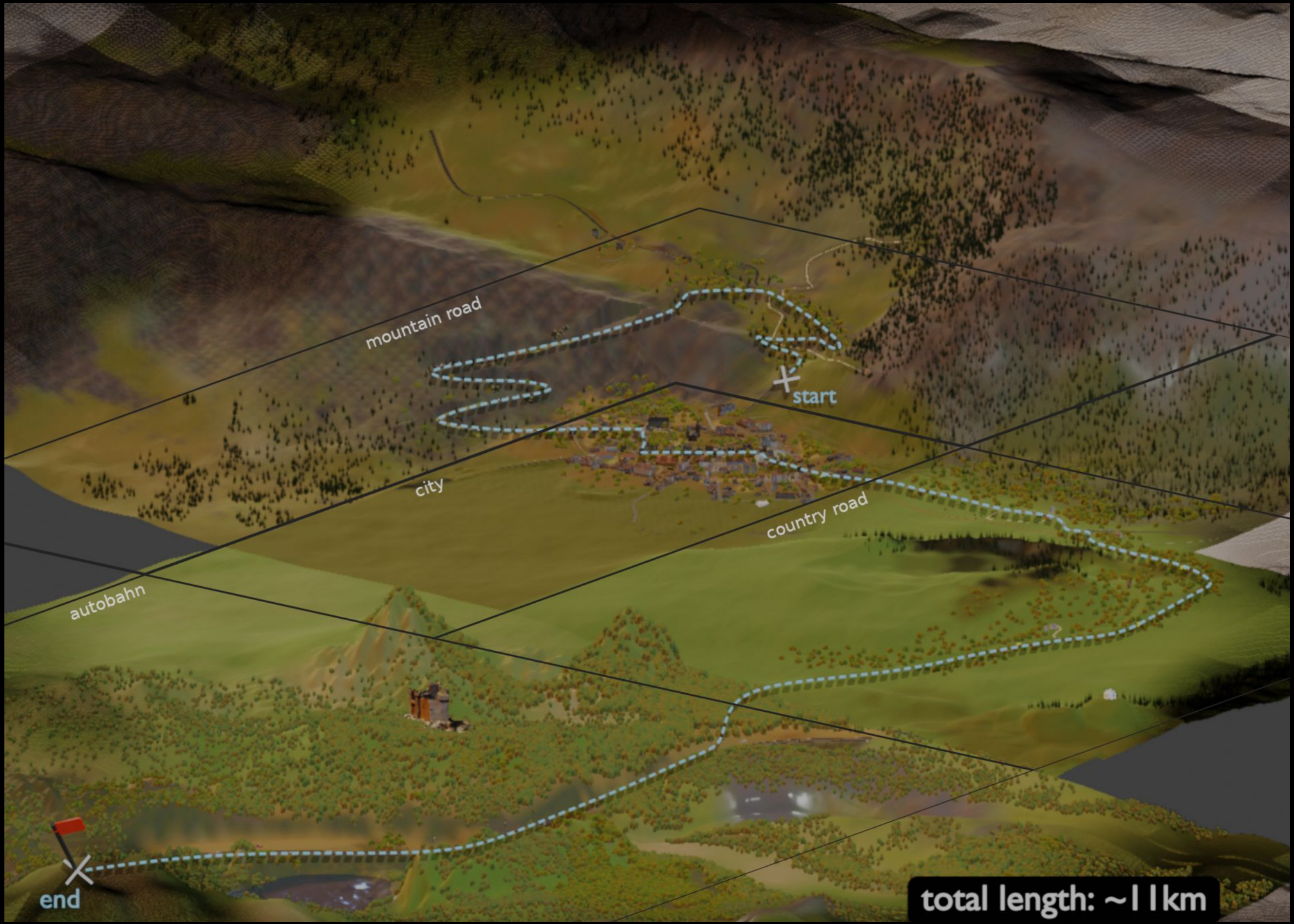

experiment map preview

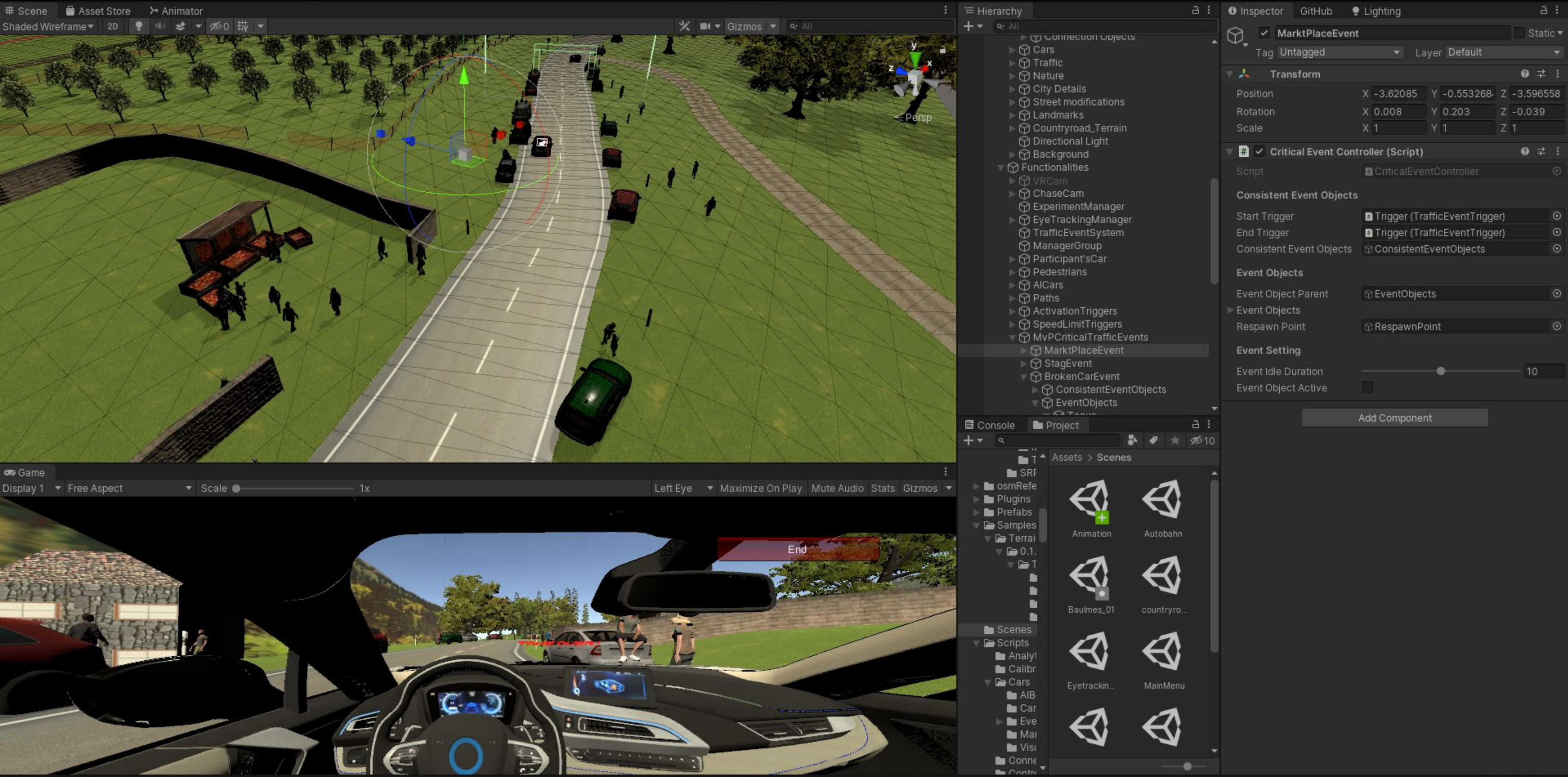

functional experiment scene (country road) in the Unity3D engine

Fully customizable in augmented reality head up display on the car’s windshield.

Highlighting the speed limit, current speed, critical event and objects of interest as well as car’s working mode.

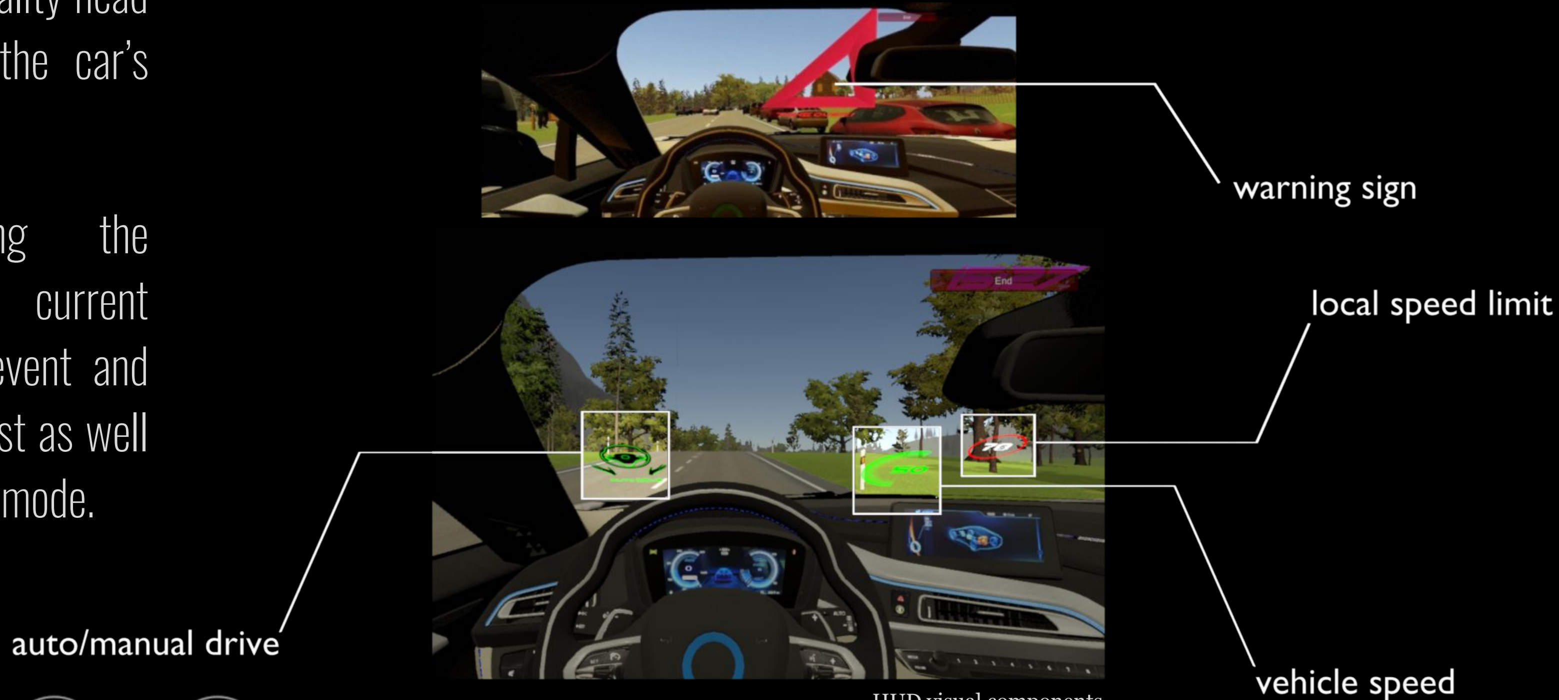

HUD visual components

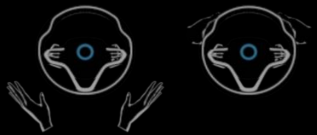

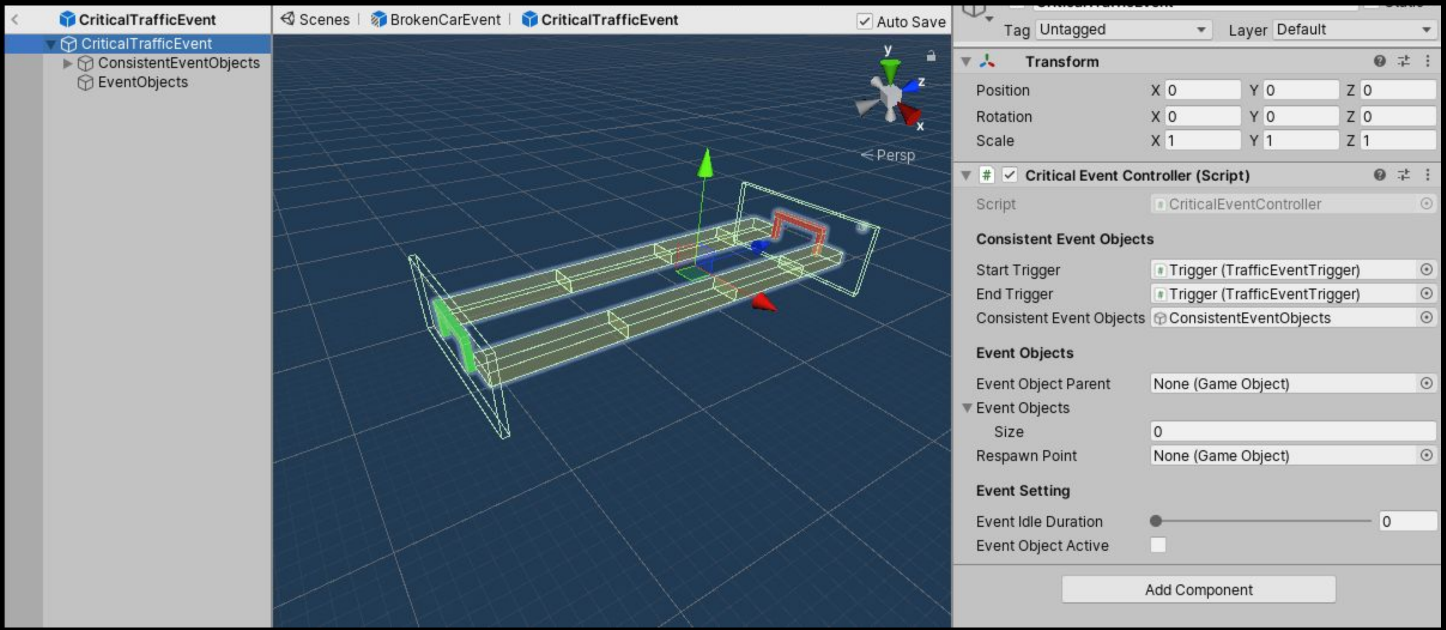

Custom prefabricated event system with editor visuals to facilitate creation of time sensitive and traffic related events.

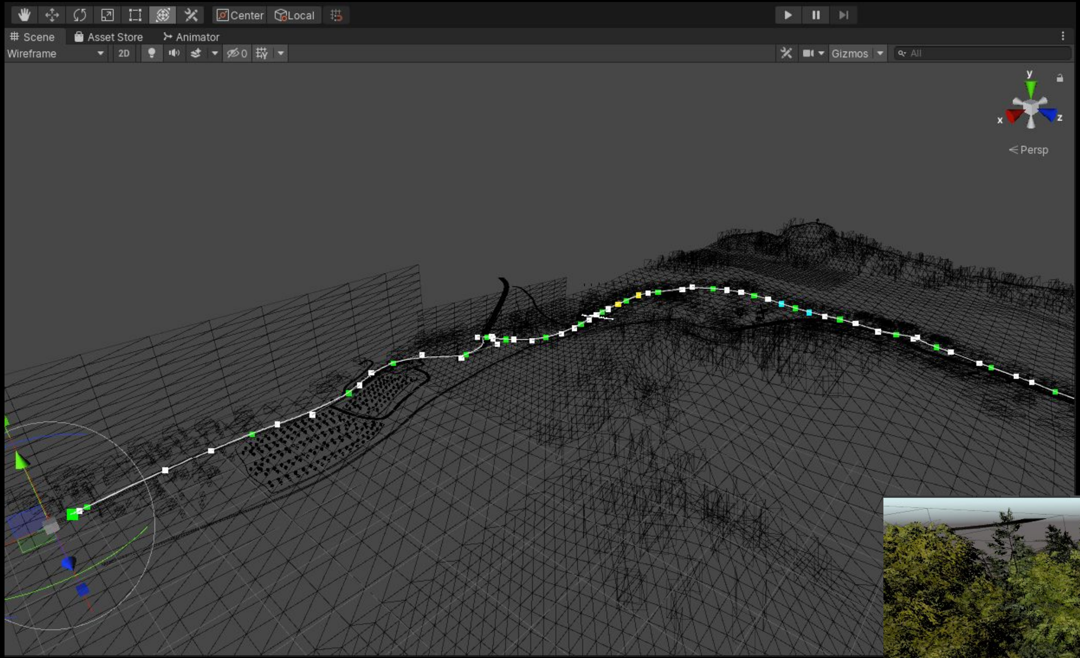

car path based on bezier curve in the Unity3D engine

Bezier curve path creation system with full control on nodes to create custom path for dynamic objects including cars, pedestrians and event objects

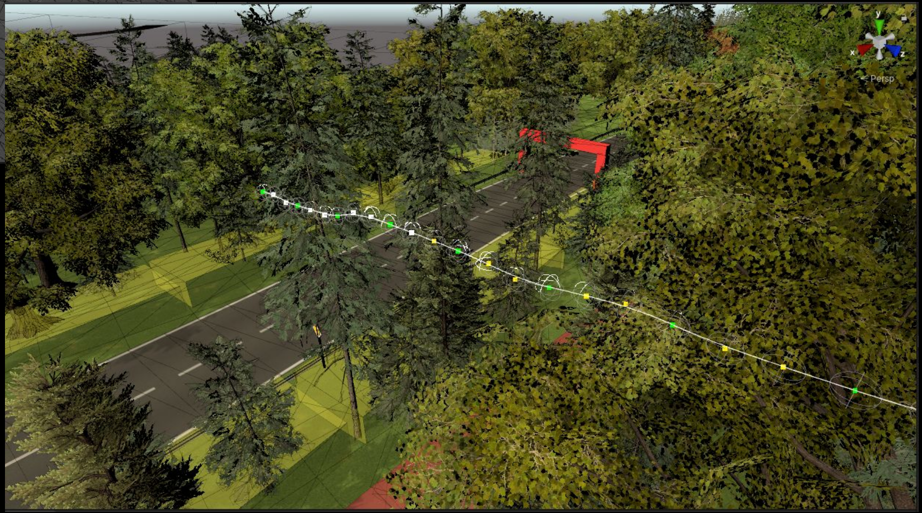

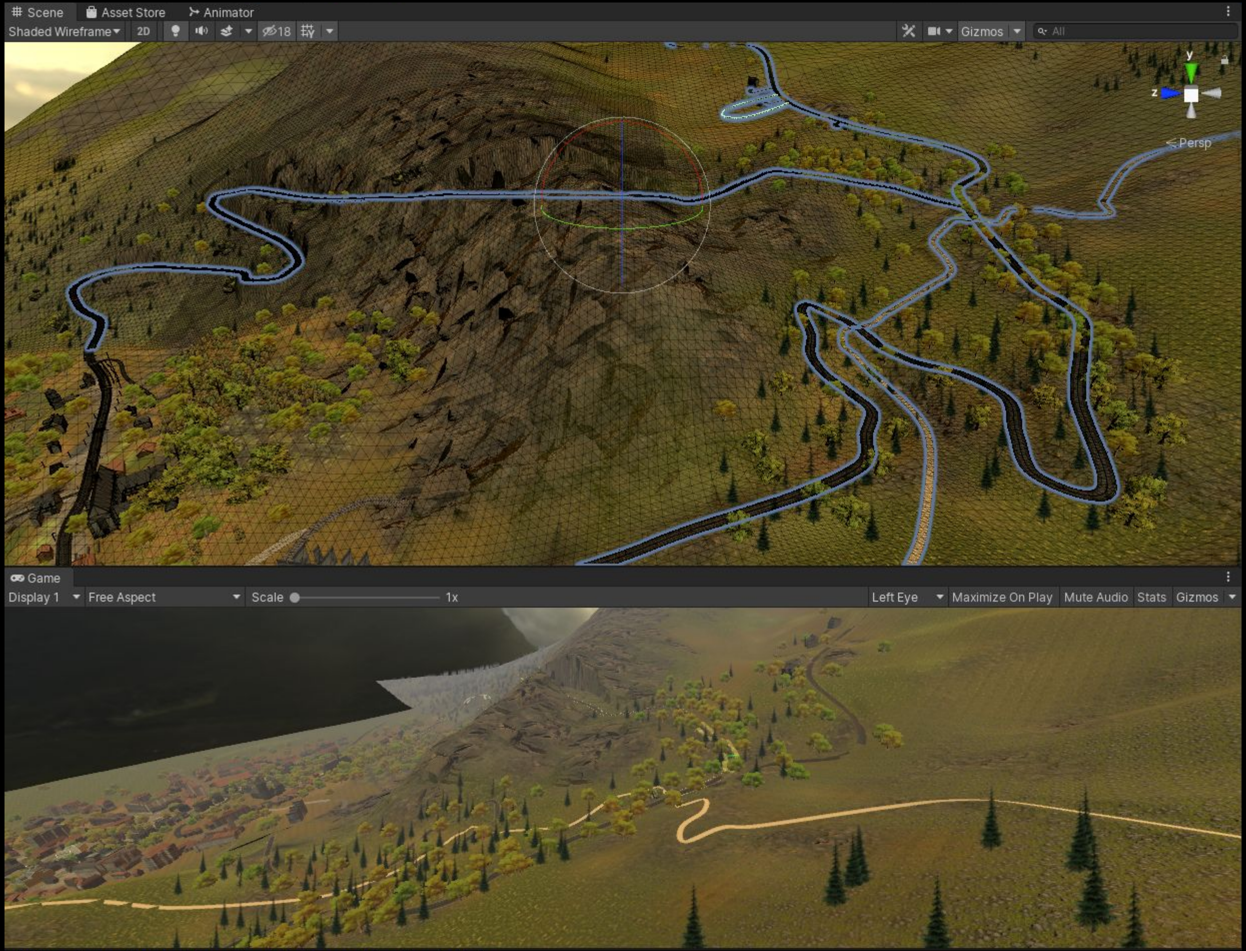

the mountain road scene in Unity3D engine

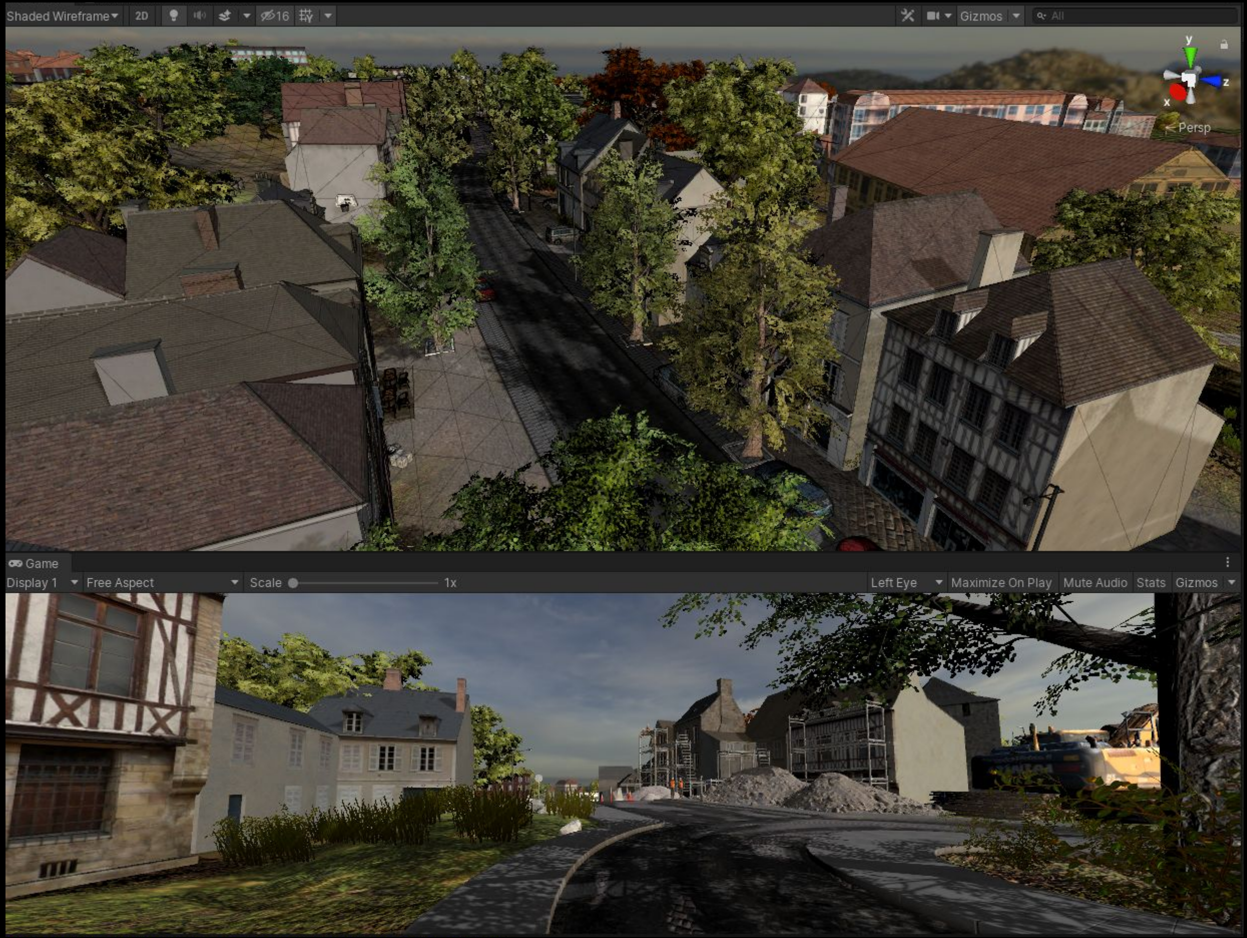

the city scene in Unity3D engine

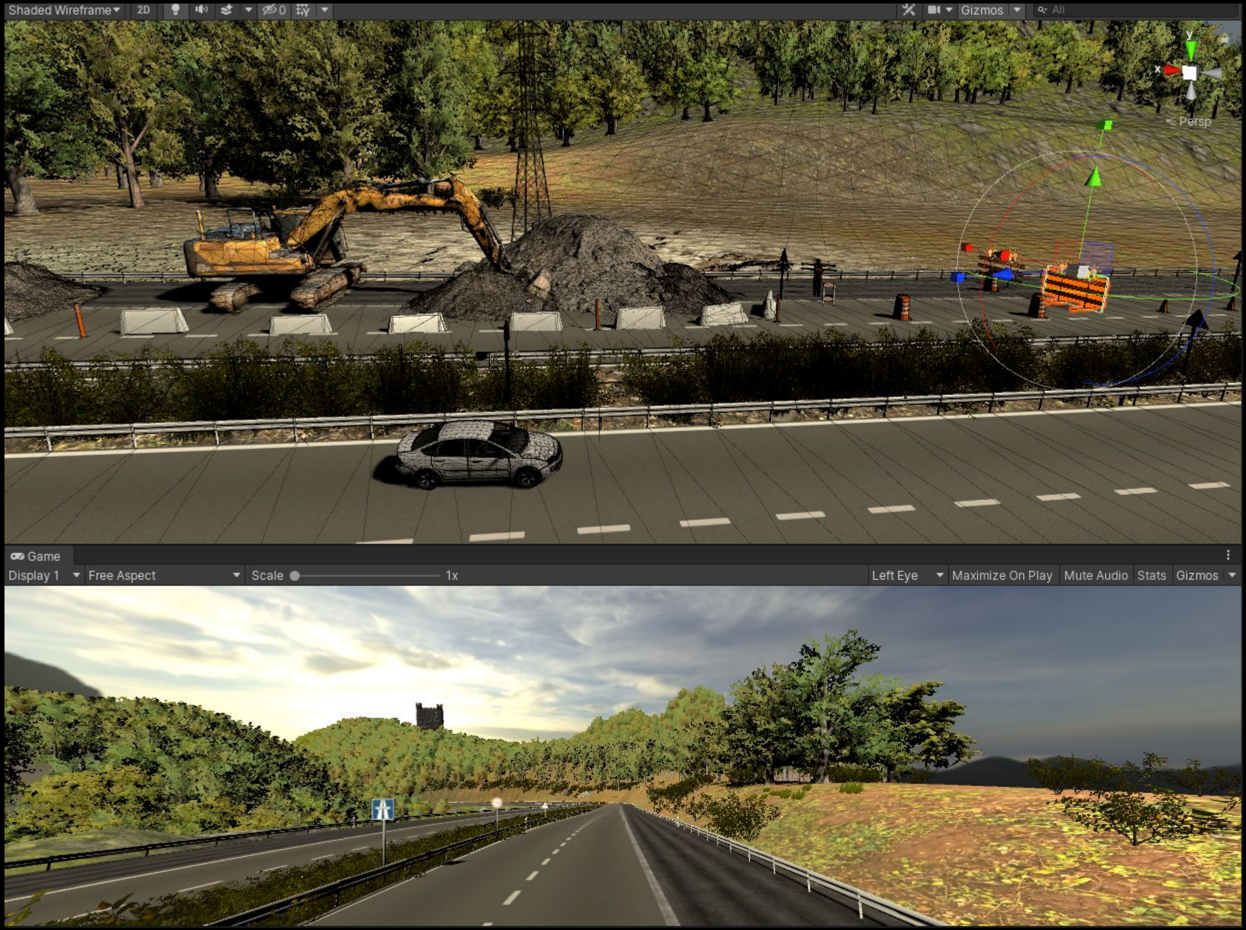

the autobahn scene in Unity3D engine

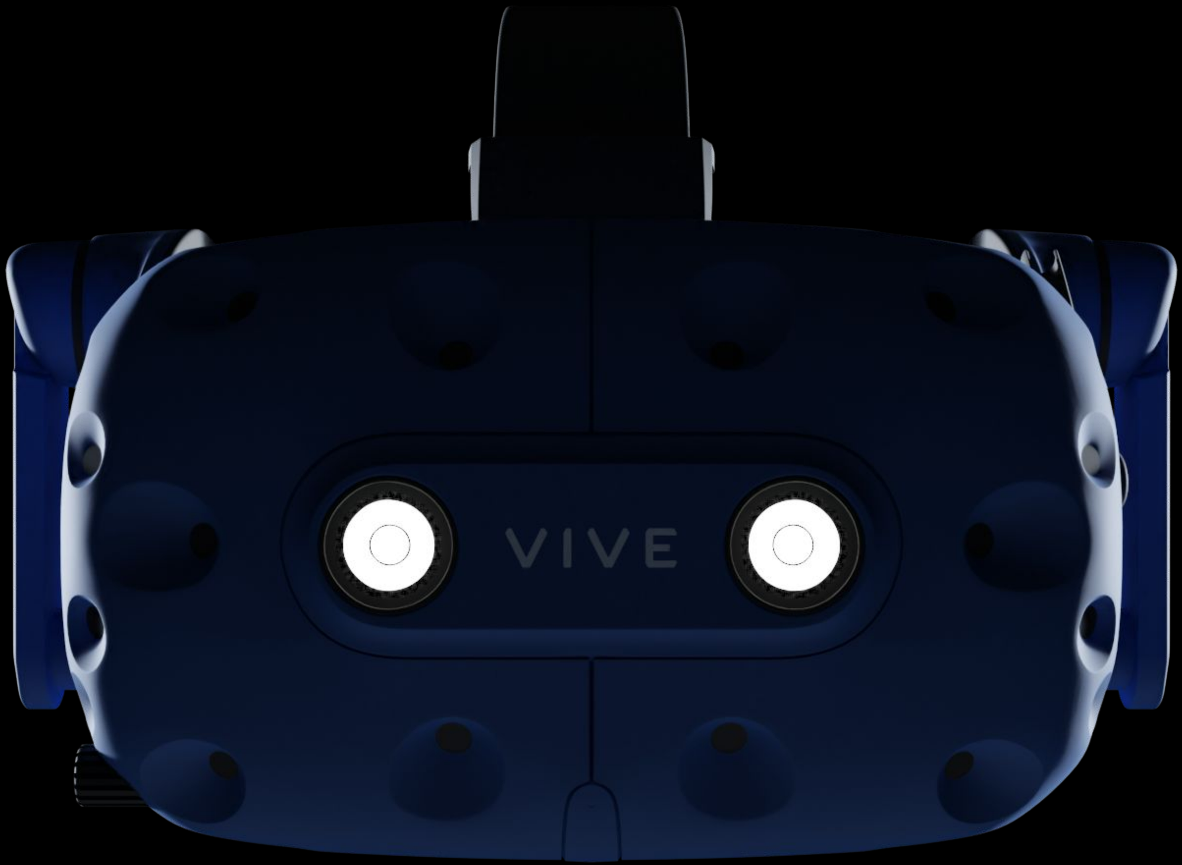

For future VR research, we tried to build the LoopAR project as easy to use toolkit inside of Unity3D, to easily produce further virtual reality experiments.
